# Different acupuncture and moxibustion therapies in the treatment of IBS-D with anxiety and depression: A network meta-analysis

**DOI:** 10.1097/MD.0000000000037982

**Published:** 2024-04-26

**Authors:** Yi Hou, Xiaoli Chang, Ningning Liu, Zhen Wang, Zhengwen Wang, Shaozong Chen

**Affiliations:** a School of Acupuncture and Massage, Shandong University of Traditional Chinese Medicine, Jinan, Shandong, China; b Research Institute of Acupuncture and Moxibustion, Shandong University of Traditional Chinese Medicine, Jinan, Shandong, China; c Shandong University of Traditional Chinese Medicine Affiliated Hospital, Jinan, Shandong, China.

**Keywords:** acupuncture and moxibustion, anxiety and depression, IBS-D, network meta-analysis

## Abstract

**Background:**

Currently, a variety of Western medical interventions are available for the treatment of diarrhea-predominant irritable bowel syndrome (IBS-D) with comorbid anxiety and depression. However, the attendant negative effects also emerge, putting pressure on healthcare resources and socio-economic structures. In recent years, the benefits of acupuncture (ACU) and moxibustion in the treatment of IBS-D with anxiety and depression have gradually emerged. However, there are many types of ACU-moxibustion-related treatments, and the aim of this study is to examine the effectiveness of different ACU-moxibustion therapies in the treatment of anxiety and depression in IBS-D patients.

**Methods:**

Searched and identified randomized controlled trials (RCTS) of ACU for the treatment of anxiety and depression in patients with irritable bowel syndrome (IBS). The search spanned from the establishment of the database until September 1, 2023. Revman 5.4 and Stata 15.0 software were used for network meta-analysis (NMA), and the included interventions were ranked by the area under the cumulative ranking curve.

**Results:**

A total of 26 articles involving 8 interventions were included. In terms of improving HAMA score, MOX was superior to EA, combined therapies, CH, WM and placebo; In terms of improving HAMD score, MOX was superior to ACU, EA, combined therapies, WM and placebo; In terms of improving the SAS score, The combined therapies were superior to EA, CH and WM; In terms of improving SDS scores, The combined therapies were superior to EA, CH and WM; In terms of improving IBS-SSS score, The combined therapies were superior to WM; In terms of reducing recurrence rates, CH was superior to combined therapies; In terms of improving total effective rates, MOX was superior to EA, CH, WM and placebo; MOX, combined therapies, ACU and EA ranked higher in SUCRA of different outcome indicators.

**Conclusion:**

MOX, combined therapies, ACU and EA have certain curative effect on anxiety and depression in patients with IBS-D, and their safety is high. ACU and MOX combined with other therapies also have significant advantages in the treatment effect.

## 1. Introduction

Irritable Bowel Syndrome (IBS) is classified as a functional gastrointestinal disorder, typically manifesting in the absence of discernible organic abnormalities. Its primary symptoms encompass abdominal pain, flatulence, and discomfort, often accompanied by alterations in stool frequency or consistency, such as diarrhea or constipation, and constipation. Notably, IBS stands as the most extensively investigated ailment within the realm of gut-brain interaction disorders.^[1]^ The documented prevalence of IBS varies between 2% and 15% in Western or Asian nations, with a tendency for younger individuals to be affected in Asian populations.^[2]^According to Rome IV criteria, IBS subtypes are divided into constipation type (IBS-C), diarrhea type (IBS-D), mixed type (IBS-M) and undetermined type (IBS-U), among which IBS-D accounts for about 40% of IBS patients and is the most common subtype in IBS.^[3]^ Patients with IBS-D have a low quality of life, and frequent diarrhea and other uncomfortable symptoms can lead to social, work, and daily life distress. At the same time, IBS-D patients are also more likely to suffer from diseases related to mental states, such as depression and anxiety.^[4]^

At present, the specific pathogenesis of IBS-D is still unclear. With the in-depth research in recent years, visceral hypersensitivity, intestinal microbiota changes, psychological and physical stress, gastrointestinal motility changes, and abnormal brain-gut interaction are considered to be the possible factors leading to IBS-D.^[5]^ Most IBS-D patients have a high level of anxiety or depression, and 23% of them have anxiety and depression.^[6]^ Clinical treatment is often based on intestinal modulators and antianxiety and depression drugs, such as 5-HT3 receptor antagonists (such as alosetron), selective serotonin reuptake inhibitors, selective norepinephrine reuptake inhibitors, and benzodiazepines (such as alprazolam), as well as diet and lifestyle interventions.^[7]^However, most of these methods have some shortcomings or controversies, and long-term use of such drugs is easy to produce adverse reactions such as dizziness, insomnia, ventricular tachycardia and drug resistance, and easy to reduce the compliance of patients, which cannot be ignored.^[8]^ In recent years, acupuncture (ACU) and moxibustion therapy have been increasingly recognized and accepted by patients at home and abroad due to its advantages of simple operation, good therapeutic effect and low price.^[9]^ ACU has a long history and rich experience in the treatment of gastrointestinal diseases. In addition, a large number of clinical studies have proved that ACU is safe and effective in the treatment of IBS,^[10,11]^ and evidence-based studies have also confirmed this view.^[12,13]^ ACU and moxibustion therapy can improve qi and blood function, enhance immunity and improve mental health by stimulating specific acupoints in the human body. Different ACU and moxibustion therapies can cause different reactions through different techniques and specific acupoints.^[14–16]^ Previous studies usually compare different ACU therapies in pairs with drugs or different interventions, and there is a lack of direct comparison evidence for the differences in efficacy between different types of ACU therapies. This study used network meta-analysis to analyze the differences in efficacy between different ACU and moxibustion therapies, so as to provide an evidence-based basis for clinical selection of the best treatment method.

## 2. Materials and methods

### 2.1. Registration

This meta-analysis has been registered on PROSPERO, registration number CRD42023465011.

### 2.2. Literature search

**Figure 1.**
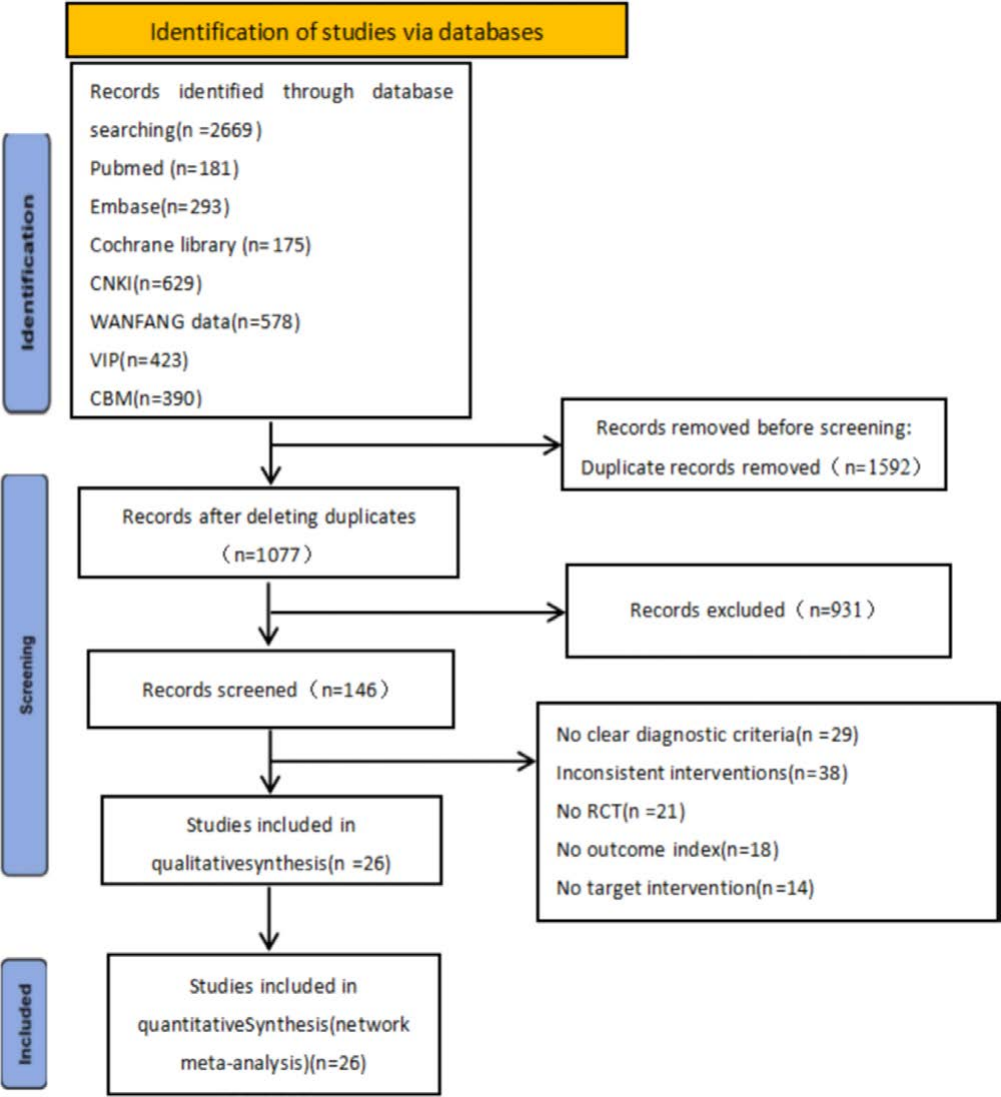
Flow diagram.

The databases of CNKI, WanFang Data, CBMdisc, PubMed, Web of Science, Cochrane Library, VIP and Embase were searched by computer. All the literature related to ACU and moxibustion in the treatment of anxiety and depression in IBS-D was collected from the establishment of the database to September 1, 2023. The retrieval methods were combined with subject words and free words and were adjusted according to different retrieval systems. Search terms: ACU-moxibustion, ACU therapy, electro ACU, ACU, moxibustion, warm ACU, Diarrhea IBS. The literature search process is shown in Figure 1. The search strategy using PubMed database as an example is included in the Supplementary File Table S1, http://links.lww.com/MD/M307, Supplemental Digital Content.

### 2.3. Literature inclusion criteria

Literature type: Randomized controlled clinical study (RCT); The object of study, has been reported in the literature of IBS-D accurate diagnostic criteria, such as Rome I to IV or expert consensus; Patients with comorbid anxiety and/or depression, meeting the diagnostic criteria of mild, moderate and severe anxiety and depression in Western medicine, such as 7 ≤ HAMA score < 29, the diagnosis is clear, without age and gender restrictions; Intervention measures: treatment group: ACU, moxibustion, warming needle and other ACU related treatment methods; The control group was treated with medication or other ACU measures different from the treatment group. Outcome indicators: Primary outcome indicators: Self-rating depression scale (SDS), Self-rating anxiety scale (SAS), Hamilton depression scale (HAMD), Hamilton Anxiety Scale (HAMA) scores; Secondary outcome measures: Total effective rate, IBS symptom severity score (IBS-SSS), recurrence rate and adverse events.

### 2.4. Literature exclusion criteria

Articles for which full text or specific data could not be obtained duplicate published articles conference reviews or abstracts related indicators of IBS-D anxiety and depression were not reported other types of IBS studies.

### 2.5. Literature screening and data extraction

Firstly, a preliminary literature review was conducted to eliminate duplicate literature. Secondly, the literature was screened according to the title and abstract. The full text assessment was then performed to screen out the RCTS that met the inclusion and exclusion criteria. Literature selection and data extraction were carried out independently by the first and second works of this paper back-to-back. After the retrieval, the included literature and relevant data extracted were cross-checked. Excel2016 was used to establish a data extraction table to extract data that met the requirements. The extracted content mainly included the first author of the literature, publication time, treatment plan, total number of participants, age and course of disease, and outcome indicators. In case of disagreement, a third-party expert will be consulted for judgment and discussion.

### 2.6. Literature quality assessment

For quality assessment, Cochrane tools were used from randomized sequence generation, allocation concealment, blinding of patients and personnel, blinding of outcome assessment, incomplete outcome data, selective outcome reporting, and other sources of bias. Using RevMan5.4, risk levels were expressed as “high risk,” “low risk,” or “unclear risk.”

### 2.7. Statistical analysis

SAS, SDS, HAMA, HDMA and IBS-SSS scores were numerical variables, and standard mean difference (SMD) was used; total effective rates and recurrence rates were categorically variable, and risk ratio (RR) was used. RevMan5.4 software was used to assess the risk of bias, and Stata16.0 software was used for frequency network Meta-analysis. network and mvmeta package commands were used for data processing, network evidence map drawing, network league table, and area under the cumulative ranking curve (SUCRA) ranking. The surface shows the SUCRA scores of all interventions, and higher SUCRA values indicate higher treatment levels. Funnel plots were generated using Stata16.0 to assess the presence of secondary sample effects in the included studies. Inconsistency factor the network evidence graph shows that there is a closed loop between the results of different studies, the ifplot command needs to be used for inconsistency detection, and the inconsistency factor value and 95% confidence interval (CI) of each closed loop need to be calculated to evaluate the consistency of the results of direct comparison and indirect comparison.

## 3. Results

### 3.1. Literature search

After the initial examination, a total of 189 relevant literatures were retrieved, including 165 Chinese literatures and 24 English literatures, 43 duplicate literatures were excluded, 93 literatures were excluded according to the title and abstract, 27 literatures were excluded after full text evaluation, and 26 literatures were finally included.^[17–42]^ See Figure 1 for the detailed procedures for inclusion in the literature.

### 3.2. Basic characteristics of the included studies

As shown in Table 1, a total of 26 articles were included, including 2003 IBS-D patients. Among the 26 studies, 8 interventions were identified, including moxibustion, ACU, electroacupuncture (EA), traditional Chinese medicine, warming ACU, ACU related therapies combined with other therapies (combination therapy), Western medicine, and placebo, of which 2 were 3-arm trials^[33,37]^ and the rest were 2-arm trials. The basic characteristics of the included literature are shown in Table 1. The detailed ACU methods of the included studies are shown in Supplementary File Table S2, http://links.lww.com/MD/M308, Supplemental Digital Content.

**Table 1:** Basic characteristics of the included literature.

References	Diagnostic criteria	Intervention			Control			Course	Type of outcomes
		Treatment	n	Age (Mean ± sd)/Range	Treatment	n	Age (Mean ± sd)/Range		
Zhang 2022	RomeIV	EA	30	31.03 ± 10.06	ACU	30	31.90 ± 10.34	6W	IBS-SSS, HAMA
Ma 2022	RomeIV	Combined therapies	30	33.93 ± 7.865	CH	30	31.53 ± 7.178	2W	IBS-SSS, total effective rate, HAMA
Liang 2017	RomeIII	ACU	22	46.45 ± 11.35	WM	12	50.83 ± 14.23	6W	IBS-SSS, HAMD
Li 2011	RomeIII	ACU	35	39.10 + 11.80	WM	35	37.93 + 11.45	4W	SAS, SDS, recurrence rate
Zhong 2018	RomeIII	EA	30	30.60 ± 13.35	WM	30	30.22 ± 13.99	4W	SAS, SDS
Tian 2020	RomeIII	ACU	43	59 ± 2. 1	Placebo	43	64 ± 1. 3	3W	HAMA, HAMD, total effective rate
Shu 2018	Expert consensus	WA	30	43.26 + 12.08	WM	25	43.26 + 12.08	4W	SAS, SDS, total effective rate
Sun 2021	Rome IV	ACU	36	39 ± 10	EA	37	41 ± 10	4W	IBS-SSS, HAMD, total effective rate
Li 2018	RomeIII	MOX	30	44.27 + 11.95	WM	30	44.17 + 13.78	4W	SAS, SDS, total effective rate, recurrence rate
Huang 2019	RomeIII	MOX	29	30.75 ± 8.79	ACU	28	31.42 ± 9.35	4W	HAMA, HAMD, total effective rate
Han 2019	RomeIII	Combined therapies	40	42.05 ± 8.87	CH	40	41.72 ± 8.03	4W	SAS, SDS, total effective rate
Meng 2019	RomeIV	ACU	35	39.3 ± 11.5	WM	35	38.4 ± 13.5	4W	SDS IBS-SSS total effective rate
Zhou 2014	RomeIII	Combined therapies	45	39.3	WM	45	38.9	8W	HAMA HAMD total effective rate
Chen 2012	RomeIII	EA	34	41. 90 ± 10. 01	WM	30	40. 50 ± 8. 75	4W	HAMA HAMD total effective rate recurrence rate
Han 2013	RomeIII	Combined therapies	144	41 ± 11.12	WM	72	40 ± 10. 72	8W	SAS total effective rate
Liao 2020	RomeIV	MOX	35	37.91 ± 2.92	WM	35	38.46 ± 3	2W	SAS SDS total effective rate recurrence rate
Yang 2020	RomeIII	Combined therapies	20	54.0 ± 6.3	WM	20	54.0 ± 6.1	2W	HAMA HAMD total effective rate
		ACU	20	55.0 ± 5.4					
Chen 2021	RomeIV + Expert consensus	Combined therapies	31	41 ± 6	WM	30	39 ± 7	6W	HAMA HAMD IBS-SSS total effective rate recurrence rate
Cheng 2023	RomeIV	Combined therapies	30	45.4 ± 8.1	CH	30	44.6 ± 7.9	4W	SAS, total effective rate
LI Guiying 2018	RomeIII	Combined therapies	43	41 ± 9	CH	43	41 ± 9	6W	SAS SDS total effective rate
Zhang 2022	RomeIV	Combined therapies	40	37.22 ± 9.25	WM	40	37.89 ± 8.65	4W	SAS SDS total effective rate recurrence rate
		CH	40	38.02 ± 8.97					
Bu 2020	RomeIII	Combined therapies	46	34.2 ± 5.2	WM	46	34.1 ± 5.2	5W	SAS SDS total effective rate
Li 2022	RomeIV	Combined therapies	47	37.3 ± 9.12	WM	46	40.8 ± 9.78	2W	SAS SDS IBS-SSS total effective rate recurrence rate
Sun 2022	Expert consensus	Combined therapies	40	36.2 ± 5.2	WM	40	35.8 ± 5.5	5W	HAMA HAMD IBS-SSS total effective rate
Jia 2022	RomeIV	Combined therapies	30	37.80 ± 11.94	CH	30	38.83 ± 12.37	4W	SAS SDS total effective rate
Wei 2023	RomeIV	Combined therapies	43	35.62 ± 5.59	WM	43	(35.43 ± 5.71)	4W	HAMA HAMD IBS-SSS total effective rate

ACU = acupuncture, CH = Chinese herb medicine, combined therapies = the combination of acupuncture-related therapies and other therapies, EA = electroacupuncture, MOX = moxibustion, placebo = and placebo, WA = warm acupuncture, WM = Western medicine of anti-diarrheal or anti-spasmodic acupuncture/blank control.

### 3.3. Quality evaluation of included literature

**Figure 2.**
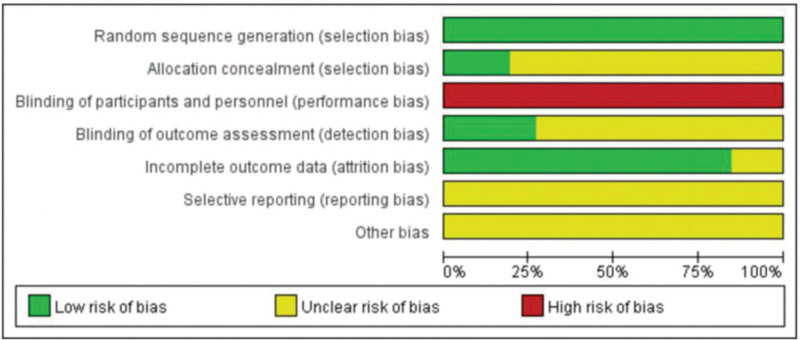
Risk of bias graph.

**Figure 3.**
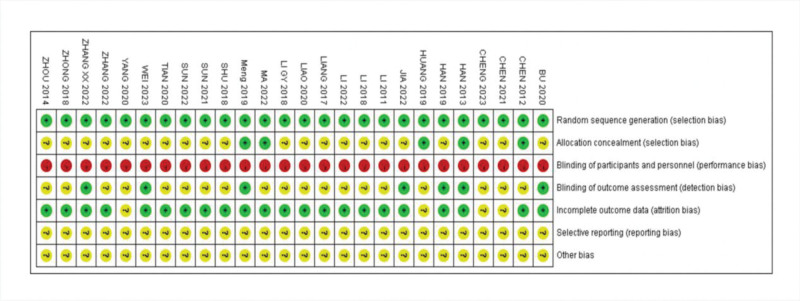
Risk of bias summary.

Twenty studies used a random number table method for randomization, and the remaining 6 studies mentioned randomization but did not specify the randomization method in the article. Due to the particularity of ACU and moxibustion operation, it is difficult to implement blinding during operation. There is no study report allocation hiding and blinding. Three studies reported missing data and were at low risk for completeness. The risk of bias was assessed in Figures 2 and 3.

### 3.4. Network meta-analysis results

#### 3.4.1. HAMA score

**Figure 4.**
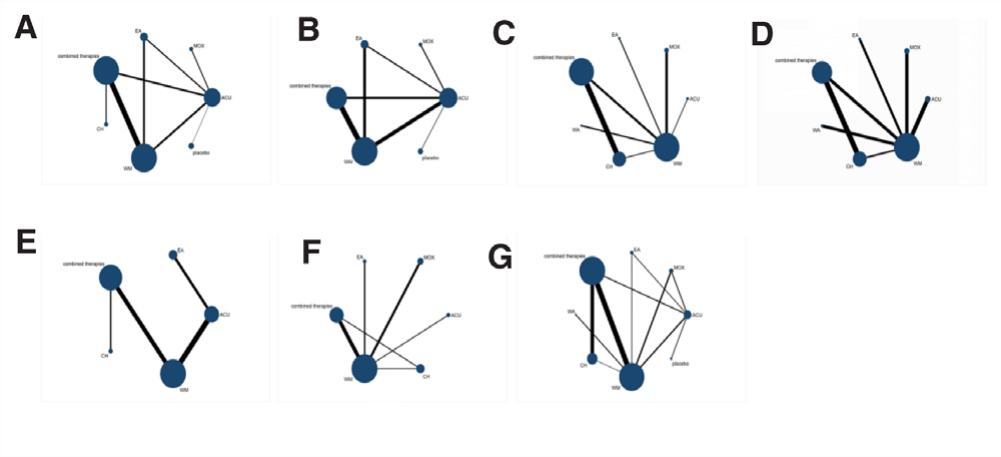
The network structure for treatment comparisons.

**Figure 5.**
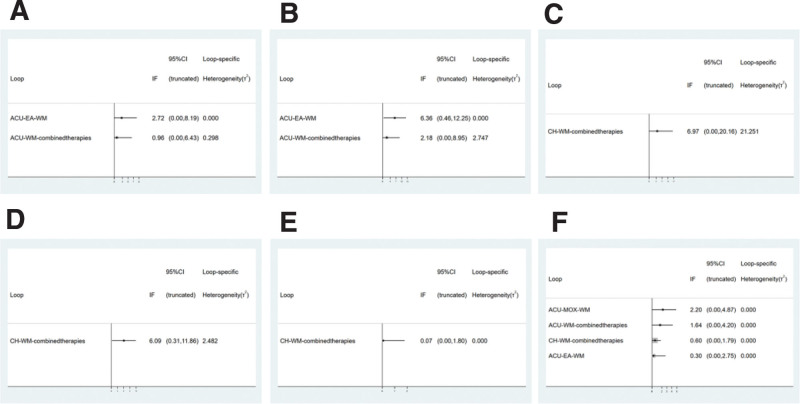
Inconsistency test.

**Figure 6.**
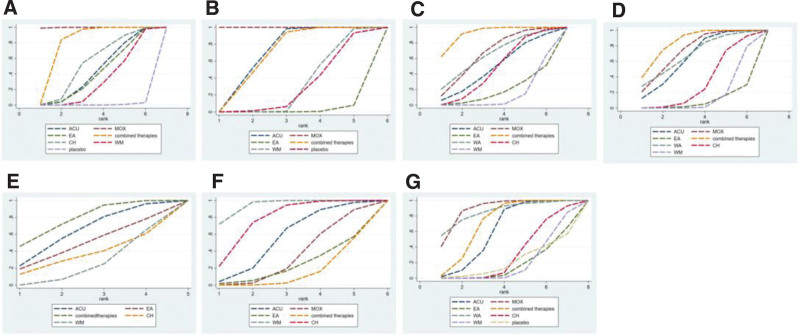
Plot of cumulative ranking probability.

**Figure 7.**
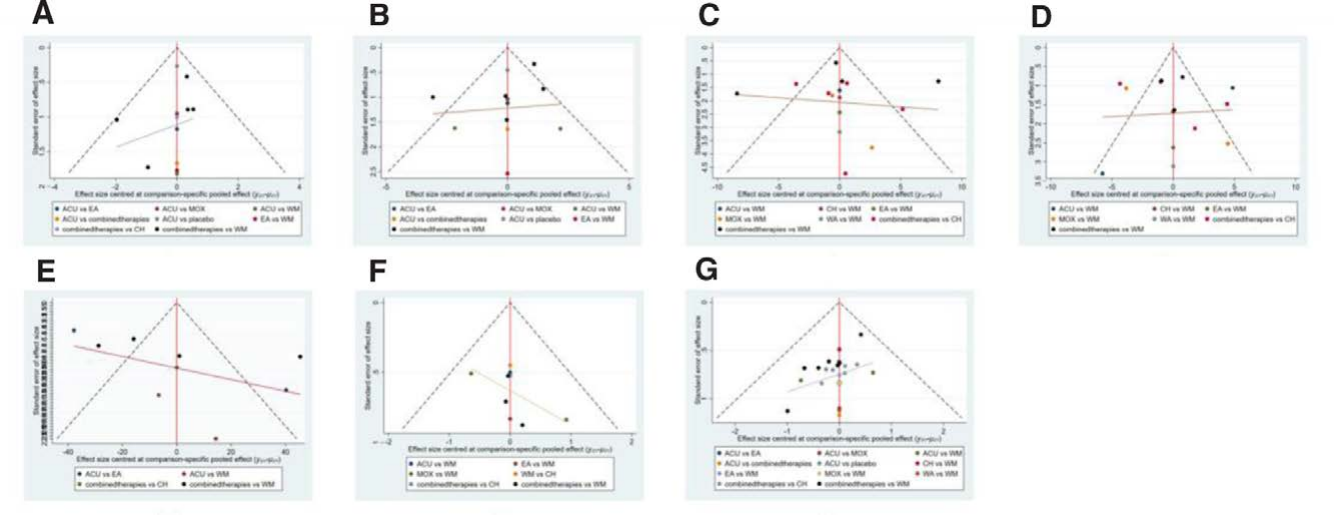
Comparison-corrected funnel plot.

A total of 10 studies mentioned HAMA scores, involving 7 interventions and 679 patients. In the network evidence plot, the connected lines represent the direct comparison between the 2 interventions, the thickness of the lines is proportional to the 2 treatments, and the size of the dots represents the sample size of different interventions. See Figure 4A. Here, we could find that after the consistency test (Fig. 5A), Seven different interventions formed 2 closed loops, namely “EA-WM-ACU”[IF = 2.72,95%CI(0.00,8.19)] and “combined therapies-WM-ACU” [IF = 0.96,95%CI (0.00,6.43)]. The 95%CI intervals of the 2 closed loops both included 0, and the 95%CI intervals of the 2 closed loops both included 0. The results of NMA are reliable. The results of NMA showed that a total of 21 different direct and indirect comparisons were formed for the 7 interventions, of which 12 were statistically significant. MOX was superior to EA, combined therapies, CH and WM in improving HAMA score (*P* < .05). ACU, MOX, EA, combined therapies, CH and WM were superior to placebo (*P* < .05). Combined therapies were superior to WM (*P* < .05) (Table 2). SUCRA probability ranking results show that, MOX (SUCRA = 99.8%) >combined therapies (SUCRA = 80.4%)>CH (SUCRA = 53.8%) > ACU (SUCRA = 43.3%) > EA (SUCRA = 40.6%) > WM (SUCRA = 31.5%) > placebo (SUCRA = 0.6%) (Fig. 6A). See Figure 7A, the “comparative-corrected” funnel plot of HAMA scores was more symmetrical, and the possibility of publication bias of the included literature was less.

**Table 2:** Network meta-analysis of HAMA scores.

ACU						
−5.65 (−7.51, −3.79)*	MOX					
0.13 (−1.93,2.18)	5.78 (3.00,8.55)*	EA				
−2.05 (−4.49,0.39)	3.60 (0.53,6.67)*	−2.18 (−4.76,0.41)	Combined therapies			
−0.62 (−3.75,2.52)	5.03 (1.38,8.68)*	−0.75 (−4.00,2.51)	1.43 (−0.54,3.40)	CH		
0.45 (−1.97,2.87)	6.10 (3.04,9.15)*	0.32 (−2.23,2.87)	2.50 (1.86,3.13)*	1.07 (−1.01,3.14)	WM	
3.07 (2.55,3.59)*	8.72 (6.78,10.66)*	2.94 (0.82,5.06)*	5.12 (2.62,7.61)*	3.69 (0.51,6.87)*	2.62 (0.15,5.10)*	Placebo

For comparison between 2 interventions,

* *P* < .05.

ACU = acupuncture, CH = Chinese herb medicine, EA = electroacupuncture, MOX = moxibustion, placebo = placebo acupuncture/blank control, WM = Western medicine of anti-diarrheal or anti-spasmodic.

#### 3.4.2. HAMD score

A total of 10 studies mentioned HAMD score, involving 667 patients and 6 interventions including ACU, MOX, EA, combined therapies, WM and placebo (Fig. 4B). Here, we could find that after the consistency test (Fig. 5B), Six different interventions formed a total of 2 closed loops. Among them, the 95%CI of “combined therapies-WM-ACU” [IF = 2.18,95%CI (0.00,8.95)] closed loops included 0, indicating that there was no obvious consistency. The 95%CI of “EA-WM-ACU” [IF = 6.36,95%CI (0.46,12.25)] closed loop did not include 0, and the minimum value was 0.46. The results of NMA showed that a total of 15 different direct and indirect comparisons were formed for the 6 interventions, of which 11 statistical comparisons were statistically significant. MOX was superior to ACU, EA, combined therapies, WM and placebo in improving HAMD score (*P* < .05). ACU was superior to EA, WM and placebo (*P* < .05). The combined therapies were superior to EA and WM (*P* < .05) (Table 3). The SUCRA probability ranking results showed that MOX (SUCRA = 100%) >ACU (SUCRA = 70.1%) >combined therapies (SUCRA = 68.3%)> WM (SUCRA = 31.0%) > placebo (SUCRA = 28.9%)>EA (SUCRA = 1.7%) (Fig. 6B). See Figure 7B, the “comparative-corrected” funnel plot of HAMD scores showed good symmetry, but some studies were distributed outside the 95%CI of the funnel plot, and there may be a small sample effect.

**Table 3:** Network meta-analysis of HAMD scores.

ACU					
−11.63 (−16.04,−7.22)*	MOX				
8.52 (4.65,12.39)*	20.15 (14.28,26.01)*	EA			
0.11 (−3.29,3.50)	11.74 (6.18,17.30)*	−8.41 (−12.94,−3.88)*	Combined therapies		
3.91 (0.81,7.01)*	15.54 (10.16,20.93)*	−4.61 (−8.89,−0.32)*	3.80 (1.88,5.73)*	WM	
4.32 (0.31,8.33)*	15.95 (9.99,21.91)*	−4.20 (−9.77,1.37)	4.21 (−1.04,9.46)	0.41 (−4.66,5.47)	Placebo

For comparison between 2 interventions,

* *P* < .05.

ACU = acupuncture, EA = electroacupuncture, MOX = moxibustion, placebo = placebo acupuncture/blank control, WM = Western medicine of anti-diarrheal or anti-spasmodic.

#### 3.4.3. SAS score

A total of 13 studies mentioned SAS score, involving 1122 patients and 7 interventions including ACU, MOX, EA, combined therapies, WA, CH and WM (Fig. 4C). Here, we could find that after the consistency test (Fig. 5C), Seven different interventions formed a closed loop, which was “CH-combined therapies-WM” [IF = 6.97,95%CI (0.00,20.16)], and the 95%CI interval included 0, indicating that the results of NMA were reliable. The results of NMA showed that a total of 21 different direct and indirect comparisons were formed for the 7 interventions, of which 4 statistical comparisons were statistically significant. MOX was superior to WM in improving SAS score (*P* < .05). The combined therapies were superior to EA, CH and WM (*P* < .05) (Table 4). SUCRA probability ranking results show that, combined therapies (SUCRA = 92.1%)> MOX (SUCRA = 66.4%)>WA (SUCRA = 64.4%)>ACU (SUCRA = 48.5%)>CH (SUCRA = 46.9%)>EA (SUCRA = 18.4%) >WM (SUCRA = 13.3%) (Fig. 6C). As shown in Figure 7C, the symmetry of the “comparison-correction” funnel plot of SAS scores was poor, and the included studies may have publication bias. In addition, some studies were distributed outside the 95%CI of the funnel plot, which may have a small sample effect.

**Table 4:** Network meta-analysis of SAS scores.

ACU						
−2.89 (−15.04,9.27)	MOX					
5.91 (−8.14,19.97)	8.80 (−3.98,21.59)	EA				
−7.55 (−18.12,3.03)	−4.66 (−13.50,4.18)	−13.46 (−24.77,−2.15)*	Combined therapies			
−2.81 (−17.42,11.80)	0.08 (−13.31,13.47)	−8.72 (−23.86,6.41)	4.74 (−7.26,16.73)	WA		
0.32 (−10.91,11.54)	3.20 (−6.41,12.82)	−5.60 (−17.52,6.33)	7.86 (3.40,12.32)*	3.13 (−9.45,15.70)	CH	
5.52 (−4.03,15.08)	8.41 (0.88,15.95)*	−0.39 (−10.72,9.94)	13.07 (8.45,17.69)*	8.33 (−2.73,19.40)	5.21 (−0.76,11.17)	WM

For comparison between 2 interventions,

* *P* < .05.

ACU = acupuncture, CH = Chinese herb medicine, EA = electroacupuncture, MOX = moxibustion, WA = warm acupuncture, WM = Western medicine of anti-diarrheal or anti-spasmodic.

#### 3.4.4. SDS score

A total of 12 studies mentioned SDS score, involving 916 patients and 7 interventions including ACU, MOX, EA, combined therapies, WA, CH, and WM (Fig. 4D). Here, we could find that after the consistency test (Fig. 5D), Seven different interventions formed a closed loop, which was “CH-combined therapies-WM” [IF = 6.09,95%CI (0.31,11.86)], the 95%CI interval did not include 0, and the minimum value was 0.31. The results of NMA showed that a total of 21 different direct and indirect comparisons were formed for the 7 interventions, of which 6 statistical comparisons were statistically significant, In terms of improving SDS score, ACU, MOX and combined therapies were superior to WM (*P* < .05). MOX was superior to EA (*P* < .05). The combined therapies was superior to EA and CH (*P* < .05) (Table 5). SUCRA probability ranking results show that, combined therapies (SUCRA = 84.6%) > MOX (SUCRA = 73.5%) > WA (SUCRA = 68.7%) > ACU (SUCRA = 65.0%)>CH (SUCRA = 33.0%) >WM (SUCRA = 16.3%)>EA (SUCRA = 9.0%) (Fig. 6D). As shown in Figure 7D, the symmetry of the “comparison-correction” funnel plot of SDS scores was poor, and the included studies may have publication bias. In addition, some studies were distributed outside the 95%CI of the funnel plot, and a small sample effect may exist.

**Table 5:** Network meta-analysis of SDS scores.

ACU						
−1.26 (−9.93,7.41)	MOX					
10.31 (−0.72,21.34)	11.57 (0.69,22.45)*	EA				
−2.87 (−10.46,4.72)	−1.60 (−8.98,5.78)	−13.17 (−23.29,−3.06)*	Combined therapies			
−0.76 (−12.26,10.75)	0.51 (−10.85,11.86)	−11.06 (−24.36,2.24)	2.11 (−8.52,12.74)	WA		
4.94 (−3.24,13.12)	6.20 (−1.83,14.23)	−5.37 (−15.96,5.22)	7.80 (3.89,11.72)*	5.69 (−5.39,16.78)	CH	
7.44 (1.23,13.66)*	8.70 (2.77,14.64)*	−2.87 (−11.98,6.25)	10.31 (5.92,14.69)*	8.20 (−1.49,17.88)	2.50 (−2.88,7.89)	WM

For comparison between 2 interventions,

* *P* < .05.

ACU = acupuncture, CH = Chinese herb medicine, EA = electroacupuncture, MOX = moxibustion, WA = warm acupuncture, WM = Western medicine of anti-diarrheal or anti-spasmodic.

#### 3.4.5. IBS-SSS score

A total of 9 studies mentioned IBS-SSS score, involving 614 patients and 5 interventions including ACU, EA, combined therapies, CH and WM (Fig. 4E). The results of NMA showed that a total of 10 different direct and indirect comparisons were formed for the 5 interventions, of which 1 statistical comparison was statistically significant, The combined therapies were superior to WM in improving IBS-SSS score (*P* < .05) (Table 6). The SUCRA probability ranking results showed that combined therapies (SUCRA = 78.0%)>ACU (SUCRA = 63.6%)>EA (SUCRA = 48.5%)>CH (SUCRA = 35.6%)> WM (SUCRA = 24.3%) (Fig. 6E). As shown in Figure 7E, the symmetry of the comparison-correction funnel plot of IBS-SSS score was poor, and the included studies may have publication bias. In addition, some studies were distributed outside the 95%CI of the funnel plot, and small sample effect may exist.

**Table 6:** Network meta-analysis of IBS-SSS scores.

ACU				
10.31 (−37.23,57.85)	EA			
−7.38 (−60.31,45.56)	−17.69 (−88.91,53.54)	Combined therapies		
26.14 (−57.33,109.61)	15.83 (−80.30,111.96)	33.51 (−33.07,100.10)	CH	
29.55 (−16.06,75.16)	19.24 (−46.68,85.16)	36.93 (4.73,69.12)*	3.41 (−69.98,76.80)	WM

For comparison between 2 interventions,

* *P* < .05.

ACU = acupuncture, CH = Chinese herb medicine, EA = electroacupuncture, WM = Western medicine of anti-diarrheal or anti-spasmodic.

#### 3.4.6. Recurrence rate

A total of 7 studies mentioned the recurrence rate, involving 488 patients and 6 interventions including ACU, MOX, EA, combined therapies, WM and CH (Fig. 4F). Here, we could find that after the consistency test (Fig. 5E), Six different interventions formed a closed loop, which was “CH-combined therapies-WM” [IF = 0.07,95%CI (0.00,1.80)], and the 95%CI interval included 0, indicating that the results of NMA were highly reliable. The results of NMA showed that a total of 15 different direct and indirect comparisons were formed for the 6 interventions, of which 4 statistical comparisons were statistically significant, In terms of reducing the recurrence rate, WM was superior to MOX, EA and combined therapies(*P* < .05); CH was superior to combined therapies(*P* < .05) (Table 7). The SUCRA probability ranking results showed that WM (SUCRA = 94.1%) >CH (SUCRA = 78.0%) >ACU (SUCRA = 55.7%) >MOX (SUCRA = 34.2%) >EA (SUCRA = 23.2%) > combined therapies (SUCRA = 14.8%) (Fig. 6F). As shown in Figure 7F, the “comparative-corrected” funnel plot of the recurrence rate was more symmetrical and less likely to have publication bias in the included literature.

**Table 7:** Network meta-analysis of recurrence rates.

ACU					
1.66 (0.45,6.06)	MOX				
2.38 (0.36,15.92)	1.44 (0.23,9.01)	EA			
2.59 (0.76,8.85)	1.56 (0.51,4.83)	1.09 (0.18,6.52)	Combined therapies		
0.43 (0.16,1.16)	0.26 (0.11,0.61)*	0.18 (0.04,0.93)*	0.17 (0.08,0.35)*	WM	
0.59 (0.16,2.16)	0.36 (0.11,1.18)	0.25 (0.04,1.56)	0.23 (0.09,0.58)*	1.37 (0.59,3.17)	CH

For comparison between 2 interventions,

* *P* < .05.

ACU = acupuncture, CH = Chinese herb medicine, EA = electroacupuncture, MOX = moxibustion, WM = Western medicine of anti-diarrheal or anti-spasmodic.

#### 3.4.7. Total effective rate

A total of 22 studies mentioned the total effective rate, involving 1776 patients and 8 interventions including ACU, MOX, EA, combined therapies, WA, WM, CH and placebo (Fig. 4G). Here, we could find that after the consistency test (Fig. 5F), Eight different interventions formed a total of 4 closed loops, Respectively as “MOX-WM-ACU” [IF = 2.20, 95% CI (0.00, 4.87)], “combined therapies-WM-ACU” [IF = 1.64, 95% CI (0.00, 4.20)], “CH-combined therapies-WM” [IF = 0.60, 95% CI (0.00, 1.79)] and “EA-WM-ACU” [IF = 0.30,95%CI (0.00,2.75)], 95%CI interval included 0, indicating that the results of NMA were highly reliable. The results of NMA showed that a total of 28 different direct and indirect comparisons were formed for the 8 interventions, of which 10 statistical comparisons were statistically significant, In terms of improving the total effective rate, ACU, MOX and combined therapies were superior to EA (*P* < .05); MOX and combined therapies were superior to CH (*P* < .05); ACU, MOX, combined therapies and WA were superior to WM (*P* < .05); MOX was superior to placebo (*P* < .05) (Table 8). SUCRA probability ranking results show that, MOX (SUCRA = 88.9%) > WA (SUCRA = 85.9%) > combined therapies (SUCRA = 71.5%) > ACU (SUCRA = 62.1%) > CH (SUCRA = 31.4%) > placebo (SUCRA = 21.5%) > WM (SUCRA = 20.4%) > EA (SUCRA = 18.2%) (Fig. 6G). As shown in Figure 7G, the “comparative-corrected” funnel plot of the total effective rate was more symmetrical, and the possibility of publication bias of the included literature was less.

**Table 8:** Network meta-analysis of total effective rates.

ACU							
0.38 (0.11,1.31)	MOX						
3.34 (1.01,11.05)*	8.72 (1.93,39.52)*	EA					
0.74 (0.29,1.90)	1.93 (0.64,5.81)	0.22 (0.07,0.75)*	Combined therapies				
0.32 (0.03,3.38)	0.83 (0.07,9.40)	0.10 (0.01,1.14)	0.43 (0.05,3.97)	WA			
2.35 (0.83,6.70)	6.14 (1.87,20.20)*	0.70 (0.19,2.59)	3.18 (1.89,5.34)*	7.41 (0.76,71.75)	CH		
2.91 (1.21,7.00)*	7.59 (2.69,21.48)*	0.87 (0.27,2.79)	3.93 (2.72,5.67)*	9.16 (1.02,82.20)*	1.24 (0.69,2.21)	WM	
3.32 (0.63,17.50)	8.68 (1.10,68.48)*	1.00 (0.13,7.71)	4.49 (0.67,30.31)	10.47 (0.58,188.21)	1.41 (0.20,10.07)	1.14 (0.17,7.48)	Placebo

For comparison between 2 interventions,

* *P* < .05.

ACU = acupuncture, CH = Chinese herb medicine, EA = electroacupuncture, MOX = moxibustion, placebo = placebo acupuncture/blank control, WA = warm acupuncture, WM = Western medicine of anti-diarrheal or anti-spasmodic.

### 3.5. Occurrence of adverse reactions

Among the included studies, only 2 studies^[18,39]^ reported the occurrence of adverse reactions, both of which occurred during the treatment, one of which was mild subcutaneous, and the symptoms disappeared after starting and pressing the needle, and the other was abdominal pain and constipation during the treatment, and the symptoms disappeared after symptomatic treatment.

## 4. Discussion

With the increase of work and life pressure and the change of eating habits, the incidence of IBS-D is increasing year by year, and the accompanying anxiety and depression state has attracted wider attention.^[43]^ In recent years, the reports of ACU and moxibustion in the treatment of IBS-D with anxiety and depression have increased significantly, and it has been gradually accepted by patients at home and abroad due to its good therapeutic effect.^[44]^ Although a large number of previous clinical studies have been conducted, indirect comparisons between different ACU interventions are lacking. This study used network meta-analysis to indirectly compare different interventions and calculate the best probability. At the same time, HAMA, HAMD, SAS, SDS, IBS-SSS, recurrence rate and total effective rate were selected to comprehensively evaluate the improvement degree of different ACU and moxibustion therapies. Based on the previous network meta-analysis,^[45]^ The recurrence rate, IBS-SSS and recurrence rate were added to provide more evidence for clinical treatment.

A total of 26 articles were included in this study, involving 8 interventions, including moxibustion, ACU, EA, traditional Chinese medicine, warming ACU, ACU related therapy and other therapies (combined therapy), Western medicine and placebo. The results showed that MOX, combined therapies and CH were more effective in improving HAMA scores. MOX, ACU and combined therapies were more effective in improving HAMD score. In terms of improving SAS and SDS scores, combined therapies, MOX and WA were more effective. In terms of improving IBS-SSS score, the top 3 interventions were combined therapies, ACU and EA. In terms of reducing recurrence rate, the top 3 interventions were WM, CH and ACU. In terms of improving the total effective rate, the top 3 interventions were MOX, WA and combined therapies. Moxibustion, combination therapy, ACU and EA have higher SUCRA rankings in different outcome indicators, indicating that the above interventions can be used as the best interventions to improve anxiety and depression in IBS-D patients.

Anxiety and depression can be a cause or a consequence of IBS-D. Symptoms of IBS-D, such as diarrhea, abdominal pain and discomfort, and distress with disease control, may contribute to the exacerbation of anxiety and depressive symptoms. On the other hand, long-term anxiety and depression, especially the psychological stress associated with IBS-D, may affect the operation and sensitivity of the intestine, and also affect the quality of life and treatment effect of patients.^[46–48]^ Some studies have pointed out that ACU can promote blood circulation and increase blood supply to the intestine and brain.^[10,49]^ In addition, ACU may regulate mood and intestinal function by regulating endocrine functions, such as promoting the secretion of neurotransmitters such as β-endorphins, dopamine, and serotonin.^[50,51]^ Moreover, a growing number of studies support the potential of ACU in the treatment of anxiety and depression. ACU may promote brain neural plasticity, change the functional connectivity and structure of the brain, and then affect emotion regulation and cognitive function. Such changes in neural plasticity may help relieve anxiety and depression symptoms.^[52,53]^ It regulates the ratio of sympathetic and parasympathetic activity, thereby reducing anxiety and depressive symptoms and improving mental health.^[54]^ This provides a possible mechanism for ACU to treat IBS.

This dual focus on adverse effects and safety is critical to the overall evaluation of any therapeutic intervention, which is one of the key steps to ensure that the patient benefits are maximized and the risks are minimized. Especially for long-term or complex treatment interventions, safety assessment is more important. Adverse effects may negatively affect the patient quality of life and may lead to treatment interruption or switch to other treatment options. Therefore, timely detection and evaluation of adverse reactions is essential to protect the health of patients. In many cases, the efficacy and safety of a treatment are not a simple trade-off between the 2, but rather a complex balancing process. A treatment may be highly effective in some patients but may present a safety risk in others. Therefore, these 2 factors need to be considered together to develop the best treatment plan. This study also has some limitations: Lack of blinding: in ACU treatment, it is difficult to achieve complete blinding because both therapists and subjects can perceive the manipulation of ACU. The lack of blinding may have introduced interference from treatment and expectation effects, thus affecting the interpretation and comparison of results. There were few detailed descriptions of randomization and allocation concealment, and the research was not deep enough. Most of the included articles were published in Chinese journals, which may have publication bias. Insufficient follow-up and lack of observation of the long-term effect of treatment. In view of the limitation of the available data, the combination of the 2 therapies (one of which is ACU related therapy) is unified as combined therapies, which are generally based on ACU + CH, ACU + WM, ACU + MOX therapies. The results show that, combined therapies have good therapeutic effects in improving various indicators, but there are many types of combined therapies, which combination therapy has the best effect is not analyzed in detail in this paper, and will be further elaborated in the follow-up research work.

## 5. Conclusions

Although the results show that different ACU therapies can effectively improve IBS-D with anxiety and depression, more high-quality studies are needed, especially large sample size, multi-center and rigorously designed RCTS with long-term follow-up results are needed to further confirm and promote these findings.

## Author contributions

**Conceptualization:** Zhen Wang.

**Investigation:** Yi Hou.

**Methodology:** Ningning Liu.

**Resources:** Xiaoli Chang.

**Software:** Xiaoli Chang.

**Validation:** Zhen Wang.

**Visualization:** Zhengwen Wang.

**Writing – original draft:** Shaozong Chen.

**Writing – review & editing:** Yi Hou.

## Supplementary Material





## References

[1] Tang HY Jiang AJ Wang XY. Uncovering the pathophysiology of irritable bowel syndrome by exploring the gut-brain axis: a narrative review. Ann Transl Med. 2021;9:1187. 34430628 10.21037/atm-21-2779 PMC8350700

[2] Choung RS Locke GR 3rd. Epidemiology of IBS. Gastroenterol Clin North Am. 2011;40:1–10. 21333897 10.1016/j.gtc.2010.12.006

[3] Drossman DA Hasler WL. Rome IV-functional GI disorders: disorders of gut-brain interaction. Gastroenterology. 2016;150:1257–61. 27147121 10.1053/j.gastro.2016.03.035

[4] Howell CA Kemppinen A Allgar V. Double-blinded randomised placebo controlled trial of enterosgel (polymethylsiloxane polyhydrate) for the treatment of IBS with diarrhoea (IBS-D). Gut. 2022;71:2430–8. 35760493 10.1136/gutjnl-2022-327293 PMC9664110

[5] Mayer EA Ryu HJ Bhatt RR. The neurobiology of irritable bowel syndrome. Mol Psychiatry. 2023;28:1451–65. 36732586 10.1038/s41380-023-01972-w PMC10208985

[6] Tarar ZI Farooq U Zafar Y. Burden of anxiety and depression among hospitalized patients with irritable bowel syndrome: a nationwide analysis. Ir J Med Sci. 2023;192:2159–66. 36593438 10.1007/s11845-022-03258-6

[7] Nee J Lembo A. Review article: current and future treatment approaches for IBS with diarrhoea (IBS-D) and IBS mixed pattern (IBS-M). Aliment Pharmacol Ther. 2021;54(Suppl 1):S63–74. 34927757 10.1111/apt.16625

[8] Camilleri M Boeckxstaens G. Dietary and pharmacological treatment of abdominal pain in IBS. Gut. 2017;66:966–74. 28232472 10.1136/gutjnl-2016-313425

[9] Billings W Mathur K Craven HJ. Potential benefit with complementary and alternative medicine in irritable bowel syndrome: a systematic review and meta-analysis. Clin Gastroenterol Hepatol. 2021;19:1538–53.e14. 32961342 10.1016/j.cgh.2020.09.035 PMC8112831

[10] Qi LY Yang JW Yan SY. Acupuncture for the treatment of diarrhea-predominant irritable bowel syndrome: a pilot randomized clinical trial. JAMA Netw Open. 2022;5:e2248817. 36580333 10.1001/jamanetworkopen.2022.48817 PMC9856830

[11] Wang Z Xu M Shi Z. Mild moxibustion for irritable bowel syndrome with diarrhea (IBS-D): a randomized controlled trial. J Ethnopharmacol. 2022;289:115064. 35114338 10.1016/j.jep.2022.115064

[12] Dai YQ Weng H Wang Q. Moxibustion for diarrhea-predominant irritable bowel syndrome: a systematic review and meta-analysis of randomized controlled trials. Complement Ther Clin Pract. 2022;46:101532. 35051805 10.1016/j.ctcp.2021.101532

[13] Wei X Wen Y Wei Y. External therapy of traditional Chinese medicine for treating irritable bowel syndrome with diarrhea: a systematic review and meta-analysis. Front Med (Lausanne). 2022;9:940328. 36017012 10.3389/fmed.2022.940328 PMC9396246

[14] Pang GS Chen ZL Hong J. Effect of acupuncture and moxibustion on diarrhea-predominant irritable bowel syndrome. J Acupunct Tuina Sci. 2016;14:22–5.

[15] Zhao JM Lu JH Yin XJ. Comparison of electroacupuncture and mild-warm moxibustion on brain-gut function in patients with constipation-predominant irritable bowel syndrome: a randomized controlled trial. Chin J Integr Med. 2018;24:328–35. 29752611 10.1007/s11655-018-2838-0

[16] Tan LH Li KG Wu YY. Effect of electroacupuncture at different acupoints on the expression of NMDA receptors in ACC and colon in IBS rats. Evid Based Complement Alternat Med. 2019;2019:4213928. 30854008 10.1155/2019/4213928 PMC6377955

[17] Zhang XX. Clinical effect of electroacupuncture on diarrhea-type irritable bowel syndrome. Master’s thesis. Tianjin: Tianjin University of Traditional Chinese Medicine. 2023.

[18] Ma CL. Clinical observation on the treatment of diarrhea-predominant irritable bowel syndrome-liver stagnation and spleen deficiency syndrome with acupuncture and herbal medicine. Master’s thesis. Shandong: Shandong University of Traditional Chinese Medicine. 2022.

[19] Liang SJ. Effect of Tiaoshen Jianpi acupuncture on hypothalamic-pituitary-adrenal axis in patients with diarrhea-type irritable bowel syndrome. Master’s thesis. Nanjing: Nanjing University of Chinese Medicine. 2017.

[20] Li H. Clinical effect of acupuncture for soothing the liver and strengthening the spleen in the treatment of diarrhea-type irritable bowel syndrome. Master’s thesis. Nanjing: Nanjing University of Traditional Chinese Medicine. 2012.

[21] Zhong F Cao Y Luo R. Effect of electroacupuncture at lower he-sea points on diarrhea-predominant irritable bowel syndrome. J Anhui Univ Tradit Chin Med. 2018;37:68–71.

[22] Tian W Wei J Li QQ. Effect of Lao-ten-needle combined with Wentong acupuncture on irritable bowel syndrome caused by post-stroke emotional stress disorder. Liaoning J Tradit Chin Med. 2020;47:162–5.

[23] Shu YY. Effect of warm acupuncture combined with acupuncture on defecation and mood in patients with diarrhea-predominant irritable bowel syndrome. J Taishan Med Coll. 2018;39:785–7.

[24] Sun YZ Wang SL Yu TY. Treatment of diarrhea-predominant irritable bowel syndrome with Tiaoshen acupuncture combined with electroacupuncture: a randomized controlled study. Chin Acupunct Moxibustion. 2021;41:13–6. 10.13703/j.0255-2930.20191220-k0001 33559435

[25] Li H Zhou Y Li Z. Clinical observation of umbilical moxibustion therapy in the treatment of 30 cases of diarrhea-type irritable bowel syndrome with liver stagnation and spleen deficiency. Chin J TCM. 2018;59:2034–6.

[26] Huang HQ Fang F Zhai YX. Clinical study on the treatment of diarrhea-type irritable bowel syndrome with moxibustion at governor vessel. New Tradit Chin Med. 2019;51:241–3.

[27] Han ZM Ren LL Lu F. Effect of acupuncture combined with traditional Chinese medicine on patients with diarrhea-predominant irritable bowel syndrome. Chin Gen Med. 2019;17:1911–3.

[28] Meng GJ. Acupuncture treatment for depressive symptom in diarrhea-predominant irritable bowel syndrome: a randomized controlled study. J Acupunct Tuina Sci. 2019;17:422–6.

[29] Zhou P Zeng ZH Jiang Q. Clinical study on modified Xiaoyao powder combined with acupuncture and moxibustion in the treatment of diarrhea-predominant irritable bowel syndrome. World Sci Technol-Tradit Chin Med. 2014;16:1331–5.

[30] Chen YH Chen XK Yin XJ. Comparison of the efficacy of electroacupuncture and probiotics combined with Deanxit in the treatment of diarrhea-predominant irritable bowel syndrome. Chin J Integr Tradit Chin West Med. 2012;32:594–8. 22679715

[31] Han BJ Feng LY. Effect of combined treatment of medication and acupuncture on cytokines in patients with diarrhea-predominant irritable bowel syndrome in anxiety state. J Ration Clin Use Drugs. 2013;6:121–2.

[32] Liao YS. Clinical observation of herbal-cake-separated moxibustion in the treatment of diarrhea-type irritable bowel syndrome. Master’s thesis. Nanjing: Nanjing University of Chinese Medicine. 2022.

[33] Yang M Zou R Zhang L. Clinical study on the effect of acupuncture and moxibustion on the mental and psychological status of patients with irritable bowel syndrome of liver stagnation and spleen deficiency. Hubei J Tradit Chin Med. 2020;42:51–4.

[34] Chen Q Zhou Y Zhang M. Efficacy of acupuncture combined with salt-separated moxibustion in the treatment of diarrhoe-type irritable bowel syndrome of spleen deficiency. Shanghai J Acupunct Moxibustion. 2021;40:400–5.

[35] Cheng XC Li XJ. Clinical observation on the treatment of irritable bowel syndrome with acupuncture and Chinese medicine. Henan Tradit Chin Med. 2023;43:454–8.

[36] Li GY. Clinical observation of acupuncture and moxibustion in the treatment of diarrhea-predominant irritable bowel syndrome. Shanghai J Acupunct Moxibustion. 2018;37:187–91.

[37] Zhang YX Zhao R Lv SG. Clinical study on traditional Chinese medicine combined with umbilical moxibustion in the treatment of diarrhea-type irritable bowel syndrome (spleen-stomach weakness). Chin J TCM Emerg Med. 2021;31:2107–10.

[38] Bu LY Lv DM. Effect of acupuncture for strengthening spleen and soothing liver on brain intestinal peptide, anxiety and depression in patients with diarrhea-predominant irritable bowel syndrome. Mod J Integr Tradit Chin West Med. 2020;29:1074–7.

[39] Li Y. Curative effect of acupuncture combined with ginger-partitioned moxibustion on diarrhea type irritable bowel syndrome of liver stagnation and spleen deficiency. Master’s thesis. Hubei: Hubei University of Traditional Chinese Medicine. 2023.

[40] Sun SZ Wang W Gao SG. Clinical study on acupuncture combined with Tongxie Xiaoyao decoction in the treatment of diarrhea-predominant irritable bowel syndrome. Hebei Tradit Chin Med. 2021;44:754–8.

[41] Jia XM Chen WJ Zhong Y. Clinical observation on the treatment of irritable bowel syndrome with spleen deficiency and dampness excess by acupuncture Tiaoshen Decoction combined with Shenlingbaizhu Powder. J Guangzhou Univ Tradit Chin Med. 2021;39:1573–7.

[42] Wei XJ Jin HY Yan XH. Clinical study of acupuncture and moxibustion for the treatment of diarrhea-type irritable bowel syndrome. Shaanxi Tradit Chin Med. 2023;44:245–249 + 254.

[43] Lu J Chen Y Shi L. Cognition of abdominal pain and abdominal discomfort in Chinese patients with irritable bowel syndrome with diarrhea. Biopsychosoc Med. 2023;17:31. 37684670 10.1186/s13030-023-00286-1 PMC10486005

[44] Zhang G Zhang T Cao Z. Effects and mechanisms of acupuncture on diarrhea-predominant irritable bowel syndrome: a systematic review. Front Neurosci. 2022;16:918701. 35911986 10.3389/fnins.2022.918701 PMC9334728

[45] Wang X Shi X Lv J. Acupuncture and related therapies for the anxiety and depression in irritable bowel syndrome with diarrhea (IBS-D): a network meta-analysis of randomized controlled trials. Front Psychiatry. 2022;13:1067329. 36620677 10.3389/fpsyt.2022.1067329 PMC9816906

[46] Fond G Loundou A Hamdani N. Anxiety and depression comorbidities in irritable bowel syndrome (IBS): a systematic review and meta-analysis. Eur Arch Psychiatry Clin Neurosci. 2014;264:651–60. 24705634 10.1007/s00406-014-0502-z

[47] Lin H Guo Q Wen Z. The multiple effects of fecal microbiota transplantation on diarrhea-predominant irritable bowel syndrome (IBS-D) patients with anxiety and depression behaviors. Microb Cell Fact. 2021;20:233. 34963452 10.1186/s12934-021-01720-1 PMC8715582

[48] Lu J Shi L Huang D. Depression and structural factors are associated with symptoms in patients of irritable bowel syndrome with diarrhea. J Neurogastroenterol Motil. 2020;26:505–13. 32675388 10.5056/jnm19166 PMC7547200

[49] Bao C Wu L Shi Y. Long-term effect of moxibustion on irritable bowel syndrome with diarrhea: a randomized clinical trial. Therap Adv Gastroenterol. 2022;15:17562848221075131. 10.1177/17562848221075131 PMC8874177 35222693

[50] Sun Z Wang X Feng S. A review of neuroendocrine immune system abnormalities in IBS based on the brain-gut axis and research progress of acupuncture intervention. Front Neurosci. 2023;17:934341. 36968497 10.3389/fnins.2023.934341 PMC10034060

[51] Hu P Sun K Li H. Transcutaneous electrical acustimulation improved the quality of life in patients with diarrhea-irritable bowel syndrome. Neuromodulation. 2022;25:1165–72. 35088760 10.1016/j.neurom.2021.10.009

[52] Tu CH MacDonald I Chen YH. The Effects of acupuncture on glutamatergic neurotransmission in depression, anxiety, schizophrenia, and Alzheimer’s disease: a review of the literature. Front Psychiatry. 2019;10:14. 30809158 10.3389/fpsyt.2019.00014 PMC6379324

[53] Zhang ZJ Zhang SY Yang XJ. Transcutaneous electrical cranial-auricular acupoint stimulation versus escitalopram for mild-to-moderate depression: an assessor-blinded, randomized, non-inferiority trial. Psychiatry Clin Neurosci. 2023;77:168–77. 36445151 10.1111/pcn.13512

[54] Amorim D Brito I Caseiro A. Electroacupuncture and acupuncture in the treatment of anxiety - A double blinded randomized parallel clinical trial. Complement Ther Clin Pract. 2022;46:101541. 35124475 10.1016/j.ctcp.2022.101541 PMC9760487

