# Supplementary material for: Different acupuncture and moxibustion therapies in the treatment of IBS-D with anxiety and depression: A network meta-analysis
Source: Medicine (Baltimore). 2024 Apr 26;103(17):e37982. doi: 10.1097/MD.0000000000037982 (PMC11049765; doi:10.1097/MD.0000000000037982)
Supplement: Supplementary file 1 [file medi-103-e37982-s001.docx]

Table S1:

**The search strategies for the included RCTs for Pubmed.**

#1 "irritable bowel syndrome"[MeSH Terms] OR "irritable bowel syndromes"[Title/Abstract] OR "syndrome irritable bowel"[Title/Abstract] OR "colon irritable"[Title/Abstract] OR "irritable colon"[Title/Abstract] OR "colitis mucous"[Title/Abstract] OR "colitides mucous"[Title/Abstract] OR "mucous colitides"[Title/Abstract] OR "mucous colitis"[Title/Abstract]

#2 "Diarrhea"[MeSH Terms] OR "Diarrheas"[Title/Abstract]

#3 "acupuncture therapy"[MeSH Terms] OR "acupuncture treatment*"[Title/Abstract] OR "treatment acupuncture"[Title/Abstract] OR "therapy acupuncture"[Title/Abstract] OR "acupuncture therapy"[Title/Abstract] OR "acupotom*"[Title/Abstract]

#4 (randomized controlled trial[pt] OR controlled clinical trial[pt] OR randomized[tiab] OR placebo[tiab] OR drug therapy[sh] OR randomly[tiab] OR trial[tiab] OR groups[tiab])

#5 #1 AND#2 AND#3 AND#4

**The search strategies for the included RCTs for EMBASE.**

#1'diarrhea'/exp

#2 'diarrhea, toxic':ti,ab,kw OR 'diarrhoea' OR 'diarrhoea, toxic':ti,ab,kw OR 'postoperative diarrhea':ti,ab,kw OR 'postoperative diarrhoea':ti,ab,kw OR 'scour' OR 'toxic diarrhea':ti,ab,kw OR 'toxic diarrhoea':ti,ab,kw OR 'diarrhea':ti,ab,kw

#3 #1 OR #2

#4 'irritable colon'/exp

#5 'colon spasm':ti,ab,kw OR 'colon, irritable':ti,ab,kw OR 'colonic diseases, functional':ti,ab,kw OR 'colonospasm':ti,ab,kw OR 'functional colonic diseases':ti,ab,kw OR 'irritable bowel syndrome':ti,ab,kw OR 'irritable colon syndrome':ti,ab,kw OR 'mucomembraneous colitis':ti,ab,kw OR 'mucomembranous colitis':ti,ab,kw OR 'mucous colitis':ti,ab,kw OR 'spastic colitis':ti,ab,kw OR 'spastic colon':ti,ab,kw OR 'unstable colon':ti,ab,kw OR 'irritable colon':ti,ab,kw

#6 #4 OR #5

#7'acupuncture'/exp

#8 'acupuncture therapy':ti,ab,kw OR 'shonishin':ti,ab,kw OR 'acupuncture':ti,ab,kw

#9 #7 OR #8

#10 'crossover procedure':de OR 'double-blind procedure':de OR 'randomized controlled trial':de OR 'single-blind procedure':de OR (random* OR factorial* OR crossover* OR cross NEXT/1 over* OR placebo* OR doubl* NEAR/1 blind* OR singl* NEAR/1 blind* OR assign* OR allocat* OR volunteer*):de,ab,ti

#11 #3 AND #6 AND #9 AND #10

**The search strategies for the included RCTs for Cochrane Library**

#1 MeSH descriptor: [Diarrhea] explode all trees

#2 MeSH descriptor: [Irritable Bowel Syndrome] explode all trees

#3 MeSH descriptor: [Acupuncture] explode all trees

#4 (Colon, Irritable):ti,ab,kw OR (Irritable Bowel):ti,ab,kw OR (Mucous Colitides):ti,ab,kw OR (Irritable Colon):ti,ab,kw OR (Mucous Colitis):ti,ab,kw OR (Colitis, Mucous):ti,ab,kw OR (Colitides, Mucous):ti,ab,kw OR (Irritable Bowel Syndromes):ti,ab,kw OR (Syndrome, Irritable Bowel):ti,ab,kw

#5 (Diarrheas):ti,ab,kw

#6 (Pharmacopuncture):ti,ab,kw OR (Acupuncture Treatment):ti,ab,kw OR (Treatment, Acupuncture):ti,ab,kw OR (Acupuncture Treatments):ti,ab,kw OR (Therapy, Acupuncture):ti,ab,kw

#7 (randomized controlled trial):ti,ab,kw OR (randomized):ti,ab,kw OR (controlled clinical trial):ti,ab,kw OR (randomly):ti,ab,kw

#8 #1 OR #5

#9 #2 OR #4

#10 #3 OR #6

#11 #7 AND #8 AND #9 AND #10

**The search strategies for the included RCTs for** **China National Knowledge Infrastructure (CNKI)**

(主题: 腹泻 + 腹泻型) AND (主题: 肠易激 + 肠易激综合征 + 肠易激综合症 +”肠易激综合征(腹泻型)+ 肠易激综合征腹泻) AND (主题: 针灸 + 针灸治疗 + 针灸疗法 + 针灸结合 + 针灸方法) AND (篇关摘: 随机对照试验 + 随机对照试验rcts + 随机对照试验研究+随机对照(精确))

**The search strategies for the included RCTs for Wanfang Data**

(((主题=腹泻) AND 主题=肠易激) AND 主题=针灸) AND（随机 OR 随机对照）

**The search strategies for the included RCTs for** **Chinese Scientific Journal Database (VIP)**

(((题名或关键词=腹泻 AND 题名或关键词=肠易激) AND 题名或关键词=针灸) AND 任意字段=随机)

**The search strategies for the included RCTs for China Biology Medicine (CBM)**

"腹泻"[标题:智能] AND "肠易激"[标题:智能] AND "针灸"[标题:智能] AND "随机"[全部字段:智能]
