# Supplementary material for: Different acupuncture and moxibustion therapies in the treatment of IBS-D with anxiety and depression: A network meta-analysis
Source: Medicine (Baltimore). 2024 Apr 26;103(17):e37982. doi: 10.1097/MD.0000000000037982 (PMC11049765; doi:10.1097/MD.0000000000037982)
Supplement: Supplementary file 2 [file medi-103-e37982-s002.docx]

Table S2：

Descriptions of the included acupuncture and related therapies

| References | Style of acupuncture | Names of acupuncture points used |
| --- | --- | --- |
| ZHANG 2022 | EA ACU | Tianshu(ST25),Zhongwan(RN12),Zusanli(ST36),Shangjuxu(ST37),Guanyuan(RN4) |
| MA 2022 | Combined therapies | ACU+CH:Dachangshu(BL25),Tianshu(ST25),Shangjuxu(ST37),Zusanli(ST36),Taichong(LR3),Qimen(LR14),Gongsun(SP4) |
| LIANG 2017 | ACU | Baihui(DU20),Yingtang(EX-HN3),Taichong(LR3),Zusanli(ST36),Sanyinjiao(SP6),Tianshu(ST25),Shangjuxu(ST37) |
| LI 2011 | ACU | Tianshu(ST25),Zusanli(ST36),Shangjuxu(ST37),Sanyinjiao(SP6),Taichong(LR3),Baihui(DU20),Yingtang(EX-HN3) |
| ZHONG 2018 | EA | Quchi(LI 11),Shangjuxu (ST37), |
| TIAN 2020 | ACU | Shangwan(RN13),Zhongwan(RN12),Xiawan(CV12),Qihai(RN6),Tianshu(ST25),Neiguan(PC6),Zusanli(ST36) |
| SHU 2018 | WA | Zhongwan(RN12),Dachangshu(BL25),Tianshu(ST25),Zusanli(ST36) |
| SUN 2021 | ACU EA | Baihui(DU20),Shenting(GV24),Benshen(GB13),Guanyuan(CV4),Zhongwan(RN12),Tianshu(ST25),Dachangshu(BL25),Zusanli(ST36),Shangjuxu(ST37),Hegu(LI4),Taichong(LR3) |
| LI 2018 | MOX | Shenque(RN8) |
| HUANG 2019 | MOX | Bladder meridian, Governor Vessel |
|  | ACU | Zusanli (ST36),Tianshu (ST25), Guanyuan (RN4), Zhongwan (RN12), Dachangshu (BL25), Pishu (BL20) |
| HAN 2019 | Combined therapies | ACU+CH:Shangjuxu(ST37),Tianshu(ST25),Zusanli(ST36),Taichong(LR3),Yingtang(EX-HN3),Baihui (DU20),Sanyinjiao(SP6) |
| Meng2019 | ACU | Taichong(LR3),Zusanli(ST36),Shangjuxu(ST37),Sanyinjiao(SP6),Tianshu(ST25),Baihui(DU20),Yingtang(EX-HN3), |
| ZHOU 2014 | Combined therapies | ACU+CH:Zusanli(ST36),Tianshu(ST25) |
| CHEN 2012 | EA | Baihui(DU20),Shenting(GV24),Neiguan(PC6) ,Shenmen(HT7),Zhongwan(RN12),Tianshu(ST25) ,Qihai(RN6),Sanyinjiao(SP6) ,Taichong(LR3) |
| HAN 2013 | Combined therapies | EA+CH:Tianshu(ST25),Zusanli(ST36),Pishu(BL20),Weishu(BL21),Shenshu(BL23),Dachangshu(BL25),Shangjuxu (ST37) |
| LIAO 2020 | MOX | Shenque(RN8) |
| YANG 2020 | Combined therapies | ACU+CH:Shenque(RN8),Neiguan(PC6),Tianshu(ST25),Sanyinjiao(SP6),Zusanli(ST36),Shangjuxu(ST37),Taichong(LR3),Yingtang(EX-HN3), |
|  | ACU | Neiguan(PC6),Tianshu(ST25),Sanyinjiao(SP6),Zusanli(ST36),Shangjuxu(ST37),Taichong(LR3),Yingtang(EX-HN3) |
| CHEN 2021 | Combined therapies | EA+MOX:Sishencong(EX-HN1),Shenque(RN8),Tianshu(ST25),Shuidao(ST28),Shangjuxu(ST37),Yinlingquan(SP9),Taichong(LR3),Fuliu(KI7),Neiguan(PC6) |
| CHENG 2023 | Combined therapies | ACU+CH:Ganshu(BL18),Pishu(BL20),Shenshu(BL23),Dachangshu(BL25),Zhongwan(RN12),Tianshu(ST25),Zusanli(ST36) |
| LI guiying 2018 | Combined therapies | ACU+MOX:Yingtang(EX-HN3),Baihui(DU20),Shangjuxu(ST37),Tianshu(ST25),Sanyinjiao(SP6),Zusanli(ST36),Taichong(LR3) |
| ZHANG 2022 | Combined therapies | MOX+CH:Shenque(RN8) |
| BU 2020 | Combined therapies | ACU+WM:Sanyinjiao(SP6),Tianshu(ST25),Zusanli(ST36),Taichong(LR3),Zhongwan(RN12),Shangjuxu(ST37),Baihui (DU20) |
| LI 2022 | Combined therapies | ACU+MOX:Neiguan(PC6),Tianshu(ST25),Zusanli(ST36),Shangjuxu(ST37),Sanyinjiao(SP6),Taichong(LR3),Yingtang(EX-HN3),Shenque(RN8) |
| SUN 2022 | Combined therapies | ACU+CH:Baihui(DU20),Shenting(GV24),Tianshu(ST25),Zusanli(ST36),Shangjuxu(ST37),Sanyinjiao(SP6),Taichong(LR3) |
| JIA 2022 | Combined therapies | ACU+CH:Sishencong(EX-HN1),Shenting(GV24),Yingtang(EX-HN3),Neiguan(PC6),Zusanli(ST36),Sanyinjiao(SP6),Zhongwan(RN12),Tianshu(ST25),Guanyuan(CV4) |
| WEI 2023 | Combined therapies | WA+WM:Yingtang(EX-HN3),Baihui(DU20),Tianshu(ST25),Shangjuxu(ST37),Zusanli(ST36),Taichong(LR3),Sanyinjiao(SP6) |

Note: ACU, acupuncture; EA, electroacupuncture; MOX, moxibustion; CH, Chinese herb medicine; WA, warm acupuncture; combined therapies, the combination of acupuncture-related therapies and other therapies;
